# Controlled and Modified Atmospheres Combined with 1-MCP Improve Postharvest Quality and Suppress *Botrytis cinerea* in Cut Roses (*Rosa hybrida* L.)

**DOI:** 10.3390/plants15101452

**Published:** 2026-05-10

**Authors:** Ertürk İnce, Nuray Akbudak, Oktay İnce

**Affiliations:** 1 Atatürk Horticultural Central Research Institute, Yalova 77100, Türkiye; oktay.ince@tarimorman.gov.tr; 2 Bursa Uludağ University, Faculty of Agriculture, Department of Horticulture, Bursa 16059, Türkiye; nakbudak@uludag.edu.tr

**Keywords:** 1-MCP, *Botrytis cinerea*, controlled atmosphere (CA), modified atmosphere packaging (MAP), *Rosa hybrida* L., postharvest quality, chlorophyll stability, cut flowers

## Abstract

Cut roses (*Rosa hybrida* L.) are highly sensitive to postharvest conditions, often experiencing quality losses associated with declines in SPAD values (relative chlorophyll index), color instability, *Botrytis cinerea* incidence, and impaired bud opening. This study aimed to evaluate the effects of different storage atmospheres, including controlled atmosphere (CA; 10% CO_2_ + 3% O_2_ and 6% CO_2_ + 3% O_2_), normal atmosphere (NA), and modified atmosphere packaging (MAP; LDPE1 (low-permeability MAP): 25 µm, 8000 cc m^−2^ day^−1^ O_2_ permeability; LDPE2 (high-permeability MAP): 25 µm, 12,000 cc m^−2^ day^−1^ O_2_ permeability), on SPAD values, color parameters, disease incidence, and bud development in cut rose cultivars (*Rosa hybrida* L.) cvs. ‘Rhodos’ and ‘Athena’ harvested in May, June, August, and November. The experiment was conducted as a factorial completely randomized design with seven biological replicates per treatment, each consisting of a single flower. Treatments were applied in combination with 1-methylcyclopropene (1-MCP, 625 ppb) and a commercial postharvest hydrating solution (Chrysal RVB, 1 mL L^−1^) under storage conditions of 0.5 ± 0.5 °C and 80–85% relative humidity. The results indicated that CA conditions in combination with 1-MCP maintained higher SPAD values, improved color stability, and were associated with lower *Botrytis* incidence (*p* < 0.01). In addition, the low-permeability LDPE1-based MAP treatment minimized variations in hue angle (h°) and improved bud development scores, while the hydrating solution treatment promoted bud opening, particularly in cv. ‘Athena’, although its effect on disease suppression was limited. Overall, the combined application of controlled atmosphere storage and 1-MCP generally showed superior performance in maintaining postharvest quality, reducing disease incidence, and preserving the visual and physiological attributes of cut roses, with effects varying depending on cultivar and evaluated parameter.

## 1. Introduction

The global ornamental plant industry has experienced substantial growth over recent decades, reflecting increasing consumer demand and the diversification of floricultural products. The production area increased by 17.75% to 1.79 million hectares, while the production value rose by 46.44% to €65.2 billion between 2009 and 2017. As of 2018, the Netherlands accounted for approximately 48% of the global export volume of USD 22.3 billion [1]. In Türkiye, cut flower production has expanded steadily since the 1960s, with the cultivated area increasing from approximately 500 ha in 1988 to more than 1400 ha in 2022 [2,3]. A substantial proportion of this production is carried out under greenhouse conditions in the Mediterranean climatic zone [4]. Among ornamental crops, roses are one of the most economically important and widely traded cut flowers worldwide, and their market value is largely determined by postharvest longevity and visual quality [5,6].

Cut roses are highly susceptible to postharvest quality deterioration, primarily due to disruptions in water relations and physiological imbalance following harvest. Key factors limiting vase life include high respiration rates, transpiration-induced water loss, and vascular blockage, all of which impair water uptake and accelerate senescence [7,8,9,10,11]. In addition, postharvest senescence is closely associated with oxidative stress, membrane degradation, and metabolic disorganization, which collectively contribute to rapid quality loss and reduced longevity [6,12]. Although low storage temperatures can delay senescence by reducing respiration, inadequate atmospheric regulation may adversely affect cellular water balance and overall physiological stability [13,14]. Vase life is therefore governed by the interaction of genotype, preharvest conditions, and storage or transport environments, making postharvest quality maintenance particularly challenging in cut roses.

In this context, controlled atmosphere (CA) storage and modified atmosphere packaging (MAP) technologies play a key role in regulating respiratory activity and maintaining physiological balance during postharvest storage. Elevated CO_2_ levels combined with reduced O_2_ concentrations can suppress respiration and reduce water loss; however, excessive modification of atmospheric composition may induce physiological stress [15,16]. In parallel with atmospheric regulation, the accumulation of reactive oxygen species is a critical factor driving senescence, and the effectiveness of antioxidant defense systems in maintaining membrane integrity is closely associated with postharvest performance [12]. Moreover, ethylene plays an important role in regulating flower senescence under certain storage and transport conditions, providing the physiological basis for the use of ethylene inhibitors, particularly 1-methylcyclopropene (1-MCP), to delay senescence, improve water relations, and preserve postharvest quality [5,17,18,19]. In addition, postharvest hydrating solutions are commonly used to improve water uptake and reduce vascular blockage, thereby supporting quality preservation during storage and handling.

Despite these advances, comprehensive studies evaluating the combined effects of different atmospheric conditions (NA, CA, and MAP) and chemical treatments (1-MCP and postharvest hydrating solutions) on SPAD values, color stability, *Botrytis cinerea* incidence, and bud development across multiple harvest periods remain limited. In addition, gray mold caused by *B. cinerea* represents a major constraint in the postharvest handling of cut roses, particularly under unfavorable storage and transport conditions, leading to significant reductions in quality and marketability [5]. Therefore, this study aimed to investigate the effects of different harvest periods (May–November) on SPAD values, color parameters, *Botrytis* incidence, and bud development in cut rose cultivars (*Rosa hybrida* L.) cvs. ‘Rhodos’ and ‘Athena’ under controlled atmosphere conditions (10% CO_2_ + 3% O_2_ and 6% CO_2_ + 3% O_2_) and modified atmosphere packaging treatments using LDPE1 (low-permeability MAP) and LDPE2 (high-permeability MAP). In addition, the interactions of 1-MCP and hydrating solution treatments with these atmospheric environments were evaluated.

The findings of this study provide a comprehensive evaluation of postharvest quality responses in cut roses under integrated storage strategies. By linking physiological responses, disease development, and environmental conditions, the study contributes to a better understanding of the factors governing vase life and postharvest performance, and provides a scientific basis for improving storage management and reducing quality losses in the floriculture supply chain.

## 2. Materials and Methods

### 2.1. Plant Material

Cut rose cultivars (*Rosa hybrida* L.) cvs. ‘Rhodos’ (red) and ‘Athena’ (white), grown under greenhouse conditions and obtained from the same commercial producer, were used as plant material in the experiments. The flowers were harvested at different periods (May, June, August, and November). Harvesting was performed at the early opening stage, characterized by the onset of petal reflex from the tight bud stage.

Harvesting was carried out during the early morning hours, and the cut flowers were transported to the laboratory within approximately 20 min under refrigerated conditions (4 ± 1 °C). cv. ‘Rhodos’ is a red hybrid tea rose, whereas cv. ‘Athena’ is a white hybrid tea rose with light pink margins. At harvest, flowers of both cultivars were standardized to a stem length of 55–60 cm to ensure uniformity among samples. In each harvest period, both cultivars were included in the experimental evaluations.

### 2.2. Storage Conditions

Storage trials were conducted in cold rooms with identical cooling capacity and environmental control systems. Controlled atmosphere (CA) treatments were performed in a 15 m^3^ cold room equipped with a CA control system connected to airtight chambers (0.7 m^3^ volume). Two chambers were used for CA applications: one chamber was set to CA10:3 (10% CO_2_ + 3% O_2_), and the other to CA6:3 (6% CO_2_ + 3% O_2_). Gas compositions inside the chambers were monitored daily to ensure stability throughout the storage period. Normal atmosphere (NA) and modified atmosphere packaging (MAP) treatments were carried out in a separate cold room with the same temperature and relative humidity conditions to ensure comparability between treatments.

In both cold rooms, storage conditions were maintained at 0.5 ± 0.5 °C and 80–85% relative humidity. NA and MAP samples were placed on pallets and shelves to prevent direct contact with the floor and to ensure uniform air circulation. For MAP treatments, two types of low-density polyethylene (LDPE) films were used: LDPE1 (low-permeability MAP; 25 µm thickness, 8000 cc m^−2^ day^−1^ O_2_ permeability) and LDPE2 (high-permeability MAP; 25 µm thickness, 12,000 cc m^−2^ day^−1^ O_2_ permeability), both having a moisture permeability of 16 g m^−2^ day^−1^. All atmospheric conditions (NA, CA10:3, CA6:3, LDPE1, and LDPE2) were combined with three treatment levels (control, 1-methylcyclopropene (1-MCP), and a commercial postharvest hydrating solution (Chrysal RVB, 1 mL L^−1^), resulting in a total of 15 treatment combinations.

### 2.3. Pre-Treatments

As treatment materials, 1-methylcyclopropene (1-MCP; 625 ppb, EthylBloc™, AgroFresh Inc., Philadelphia, PA, USA) and a commercial postharvest hydrating solution (Chrysal RVB Clear Intensive, 1 mL L^−1^; Chrysal International B.V., Naarden, The Netherlands) were used. Flower stems were recut by 10 mm under distilled water to prevent blockage of vascular tissues, after which the samples were divided into three groups (control, 1-MCP, and hydrating solution).

Samples assigned to the hydrating solution treatment were held for 2 h at 20 °C in a solution prepared by adding 1 mL L^−1^ of the product to distilled water. For the 1-MCP treatment, the samples were exposed to 625 ppb 1-MCP in an airtight chamber with a volume of 1 m^3^ at 20 ± 1 °C for 12 h, followed by 2 h of ventilation. Control samples were maintained in distilled water under the same conditions.

Following these treatments, all samples were subjected to precooling at 4 °C and 80–85% relative humidity. Subsequently, control, 1-MCP, and hydrating solution treatments were applied in combination under normal atmosphere (NA), controlled atmosphere (CA10:3 and CA6:3), and modified atmosphere packaging (LDPE1 and LDPE2) conditions.

### 2.4. Vase Life

Stored samples were removed from cold storage at 10-day intervals (0, 10, 20, 30, 40, 50, and 60 days) and subjected to vase life evaluations. For each treatment combination and storage interval, seven biological replicates were used, with one flower representing one replicate. The flowers were placed individually in 330 mL glass vases containing 180 mL of distilled water and maintained at 21 °C, 55–60% relative humidity, and a photoperiod of 12 h light (1000 lux) and 12 h darkness. Water levels were monitored daily and replenished to compensate for evaporation losses.

Vase life was defined as the number of days from the beginning of vase conditions until the termination criteria were reached. Termination was considered when more than 50% of the petals exhibited wilting, browning, neck bending, or symptoms of *Botrytis* infection [20,21]. Vase life duration was recorded in days for each individual stem.

#### 2.4.1. Measurement Parameters

At the end of each 10-day storage period (0, 10, 20, 30, 40, 50, and 60 days), the following analyses were conducted. For each treatment combination and storage interval, seven biological replicates were used, with one flower representing one replicate:

- **SPAD values (relative chlorophyll index):** Measured on leaves using a chlorophyll meter (Konica Minolta SPAD-502 Plus, Konica Minolta Inc., Tokyo, Japan). Measurements were taken from the same leaf position on each stem, and SPAD readings at the beginning (V_0_) and end (V_e_) of vase life were performed on the same leaf as far as possible to ensure consistency and comparability.
- Color measurements (L*, a*, b*, C*, h°): Determined on sepals and petals using a colorimeter (Konica Minolta Chroma Meter CR-300, Konica Minolta Inc., Tokyo, Japan). Chroma (C*) and hue angle (h°) were calculated according to standard colorimetric equations. Raw h° values are presented as obtained from the instrument output (−180° to +180° range), and were not transformed to the 0–360° scale to preserve the original measurement characteristics.
- ***Botrytis* incidence:** Evaluated visually using a 1–5 rating scale (1 = no infection, 5 = severe infection). All assessments were conducted under consistent environmental conditions.
- **Bud development:** Evaluated using cultivar-specific ordinal scales. For cv. ‘Rhodos’, a 1–3 scale was used (1 = closed bud, 2 = partially loosened petals, 3 = partially open flower), whereas cv. ‘Athena’ was evaluated on a 1–5 scale (1 = closed bud, 2 = slightly loosened petals, 3 = partially open flower, 4 = mostly open flower, 5 = fully open flower), reflecting differences in flower morphology and opening characteristics [22]. Due to these differences, bud development data were analyzed and interpreted separately for each cultivar. All evaluations were performed by the same observer to ensure consistency and reduce subjectivity in visual scoring.

#### 2.4.2. Experimental Design and Statistical Analysis

The experiment was conducted according to a factorial completely randomized design, including atmospheric conditions (NA, CA10:3, CA6:3, LDPE1, and LDPE2) and treatment applications (control, 1-MCP, and hydrating solution). For each treatment combination and storage interval, seven biological replicates were used, with one flower representing one replicate.

All data were analyzed using JMP 13 Pro software (SAS Institute Inc., Cary, NC, USA). The effects of atmospheric conditions, treatment applications, and storage duration were evaluated using analysis of variance (ANOVA). When significant differences were detected, mean comparisons were performed using Tukey’s multiple comparison test at the 1% significance level (α = 0.01). Correlation analysis and multiple regression analyses were conducted to evaluate relationships among measured parameters.

Due to differences in scoring scales between cultivars (1–3 for ‘Rhodos’ and 1–5 for ‘Athena’), bud development data were analyzed separately for each cultivar. No direct statistical comparison between cultivars was performed for this parameter, and analyses were conducted independently to avoid bias associated with non-comparable ordinal scales.

Due to experimental constraints, not all treatment combinations were evaluated across all harvest periods. In particular, controlled atmosphere (CA10:3 and CA6:3) treatments were limited to the initial harvest period (May), whereas subsequent evaluations focused on selected treatment combinations. Therefore, missing values in the corresponding tables indicate treatments that were not evaluated rather than missing observations. Statistical analyses were performed based on the available data within each harvest period.

## 3. Results and Discussion

Although vase life was recorded for each individual stem as described in Section 2, it is not presented as a separate outcome variable in this study. The primary objective of this study was to evaluate physiological and quality-related parameters under different storage conditions. Therefore, vase performance was assessed indirectly through SPAD values, color characteristics, *Botrytis* development, and bud development, which collectively reflect postharvest performance.

### 3.1. SPAD Values (Relative Chlorophyll Index)

SPAD values are widely used as a non-destructive indicator of relative chlorophyll status and physiological condition in plant tissues. The maintenance of SPAD values in leaf and sepal tissues during vase life is associated with the preservation of water balance and overall metabolic stability. Therefore, SPAD measurements were evaluated as an indicator of physiological changes and senescence progression in cut rose tissues.

Across the four harvest periods, atmospheric conditions and treatment applications had significant effects on SPAD values (*p* < 0.01). In cv. ‘Rhodos’, SPAD values at the beginning of vase life (V_0_) ranged between 51.4 and 61.8, while values at the end of vase life (V_e_) varied between 54.3 and 60.8 depending on treatment combinations. Among the selected treatment combinations (Table 1), 1-MCP applications, particularly under controlled atmosphere conditions (CA10:3), were associated with relatively stable SPAD responses. In addition, hydrating solution (HS) treatment under LDPE2 conditions showed a positive ΔSPAD value (+4.5), indicating improved SPAD retention compared to other treatments.

In cv. ‘Athena’, SPAD values were generally lower than those observed in ‘Rhodos’, ranging between 27.9 and 47.8 at V_0_ and between 27.8 and 43.8 at V_e_ (Table 2). Treatment effects were also significant (*p* < 0.01), with 1-MCP applications generally contributing to SPAD stability under controlled atmosphere conditions. The highest positive ΔSPAD value (+2.9) was observed under LDPE2 + HS conditions, whereas the greatest decrease in SPAD (−7.3) occurred under LDPE1 + 1-MCP conditions, indicating greater variability in SPAD response under certain MAP treatments.

In both cultivars, slight increases in SPAD values were observed in specific treatment combinations. Since SPAD measurements at V_0_ and V_e_ were performed on the same leaf as far as possible, these increases may be attributed to improved leaf hydration and turgidity affecting optical measurement properties, rather than actual chlorophyll synthesis. Therefore, positive ΔSPAD values should be interpreted as relative improvements in physiological condition.

Overall, the results indicate that 1-MCP treatment contributed to maintaining SPAD stability, while hydrating solution applications supported water balance and reduced variability in SPAD responses. These findings suggest that both ethylene regulation and water status play a role in preserving physiological quality in cut roses during postharvest storage.

In both cultivars, 1-MCP treatment contributed to maintaining SPAD stability (ΔSPAD) (*p* < 0.01). In cv. ‘Rhodos’, the CA10:3 condition combined with 1-MCP was associated with relatively stable SPAD responses, whereas in cv. ‘Athena’, the LDPE2 condition combined with hydrating solution (HS) showed improved SPAD retention (Table 1 and Table 2).

Although SPAD values represent a relative chlorophyll index rather than direct chlorophyll content, they are widely used as a non-destructive proxy for assessing chlorophyll status and physiological condition in plant tissues. In this context, the observed stability of SPAD values in the present study is consistent with previous findings indicating that reduced metabolic activity under low-temperature and controlled atmosphere conditions contributes to delayed senescence and preservation of green tissues in cut flowers [23,24,25].

Similarly, 1-MCP treatments have been reported to delay senescence and support physiological stability in cut flowers [15,16], which may explain the improved SPAD retention observed in the present study. In addition, recent studies have demonstrated that postharvest treatments can significantly influence physiological and biochemical characteristics in cut rose cultivars [6].

In addition, hydrating solution (HS) treatments showed moderate effects on SPAD preservation, which may be associated with improved water uptake and reduced vascular blockage. Similar improvements in postharvest quality and vase life have been linked to enhanced water relations in cut roses [26]. Furthermore, senescence-related physiological changes have been associated with oxidative stress and antioxidant system responses, which may also influence SPAD dynamics during vase life [12].

### 3.2. Color Changes in Sepal and Petal Tissues (h° and C*)

Changes in hue angle (h°) and chroma (C*) values in both sepal and petal tissues of cvs. ‘Rhodos’ and ‘Athena’ during vase life were significantly influenced by atmosphere × treatment interactions (*p* < 0.05). Hue angle (h°) and chroma (C*) values, calculated from L*, a*, and b* coordinates, represent color tone and saturation, respectively. In general, relatively limited variation in h° and C* values was associated with better preservation of color stability, whereas greater fluctuations indicated senescence-related changes in pigment composition. Representative atmosphere × treatment combinations showing distinct responses are presented in Table 3 and Table 4.

In cv. ‘Rhodos’, decreases in h° values during vase life were associated with shifts in petal color tone, reflecting senescence-related pigment changes. Similar trends have been reported in previous studies, where color modifications during senescence were linked to changes in pigment composition and physiological degradation processes in cut roses [18,27]. Among the evaluated treatments, combinations involving 1-MCP under controlled atmosphere (CA) conditions and modified atmosphere packaging (LDPE1 and LDPE2) were associated with relatively stable h° and C* values. In addition, hydrating solution (HS) treatments showed moderate effects on maintaining color stability, which may be related to improved water balance and reduced vascular blockage. Recent studies have also demonstrated that postharvest treatments affecting physiological and biochemical status contribute to maintaining visual quality and color stability in cut roses [6,12].

In cv. ‘Athena’, the initially high h° values were consistent with the white–cream petal structure, and the gradual decline in h° during vase life indicated progressive color changes associated with senescence. Treatments involving 1-MCP, particularly under CA conditions, contributed to maintaining higher h° values and more stable color characteristics. In addition, under conditions where white coloration was better preserved, relatively higher h° and C* values (e.g., h° ≈ −69.64 and C* ≈ 18.59) were observed in petal tissues, whereas lower values (e.g., h° ≈ −34.00 and C* ≈ 4.04) were recorded in sepal tissues under certain storage conditions. These observations are consistent with previous findings indicating that low oxygen environments and controlled storage conditions can delay oxidative processes and help maintain visual quality in white-flowered species [7,9].

Variance analysis indicated that the cultivar × treatment × period interaction was statistically significant for both h° and C* parameters (*p* < 0.05), confirming that color responses depend on the combined effects of genotype, storage conditions, and postharvest treatments. In general, treatments involving 1-MCP combined with CA and LDPE-based MAP conditions were associated with improved color stability in both cultivars. Similar relationships between ethylene inhibition, physiological stability, and color preservation have been reported in previous studies [9,19,25].

Overall, the results demonstrate that atmospheric composition and postharvest treatments play a key role in maintaining pigment stability and visual quality in cut roses. In particular, combinations involving 1-MCP and modified or controlled atmosphere conditions contributed to preserving color tone in petal tissues and maintaining color balance in sepals. These findings are consistent with previous studies conducted under both regional and international conditions for *Rosa hybrida* L., highlighting the importance of integrated postharvest strategies for preserving ornamental quality.

### 3.3. Botrytis Severity (1–5 Scale) and Incidence (%)

*Botrytis cinerea* development during vase life was significantly affected by cultivar, storage atmosphere, and postharvest treatments (*p* < 0.05). Disease severity (1–5 scale) and incidence (%) exhibited distinct patterns depending on treatment combinations and storage duration (Table 5). In general, disease severity increased progressively with storage time, particularly after 30–40 days, whereas incidence varied according to both cultivar and atmospheric conditions.

In cv. ‘Rhodos’, *Botrytis* development was observed in all treatments during extended storage periods; however, combinations involving 1-MCP, particularly under controlled atmosphere conditions (CA10:3 and CA6:3), were associated with relatively lower disease severity compared to control treatments. In contrast, cv. ‘Athena’ showed lower overall disease incidence, especially during early storage periods (10–30 days), where 1-MCP and LDPE-based MAP treatments contributed to suppressing pathogen development. These results indicate clear cultivar-dependent differences in susceptibility to *Botrytis* infection.

The permeability characteristics of packaging materials also influenced disease development. In LDPE-based MAP systems, increased relative humidity within the package was associated with higher disease severity during prolonged storage (≥40–50 days), suggesting that moisture accumulation may promote fungal reactivation. In contrast, controlled atmosphere conditions combined with 1-MCP were generally associated with reduced disease progression, although complete suppression of pathogen development was not achieved in any treatment.

These findings are consistent with previous studies indicating that low temperature and controlled atmosphere conditions can reduce respiration and delay pathogen development in cut flowers [11,23]. In addition, 1-MCP treatments have been reported to influence postharvest physiological processes and may contribute to reduced disease development depending on cultivar and treatment conditions [28,29]. However, as also reported by [30,31], prolonged storage under high humidity conditions may lead to renewed pathogen activity despite initial suppression.

Variance analysis confirmed that the interaction of cultivar × treatment × storage period significantly affected both *Botrytis* severity and incidence (*p* < 0.05). Overall, lower disease severity values were observed in cv. ‘Athena’ under 1-MCP and CA-based treatments, whereas higher severity scores were recorded in cv. ‘Rhodos’, particularly under control and long-term MAP conditions. These results highlight the importance of integrating atmospheric regulation, ethylene inhibition, and humidity management strategies to effectively control *Botrytis* development during postharvest storage.

### 3.4. Bud Development Score (1–5)

Bud development is a key physiological parameter determining the postharvest performance and visual quality of cut roses during vase life. In the present study, bud opening was evaluated using cultivar-specific ordinal scales, reflecting morphological differences between cvs. ‘Rhodos’ (1–3 scale) and ‘Athena’ (1–5 scale). Due to these differences, bud development responses were analyzed separately for each cultivar. Representative treatment effects are presented in Table 6 and Table 7.

In cv. ‘Rhodos’, bud development remained limited throughout the vase life period and generally did not exceed moderate levels (scale 2–3), and no statistically significant differences were observed among treatments (Table 6), indicating a limited responsiveness of cv. ‘Rhodos’ to postharvest applications. In contrast, cv. ‘Athena’ exhibited a broader range of bud development responses within its scoring scale, with significant differences among treatments depending on storage conditions (Table 7). Treatments involving 1-MCP combined with controlled atmosphere (CA) and LDPE-based modified atmosphere packaging were generally associated with more stable bud development patterns under certain storage conditions.

Storage duration also influenced bud development. A gradual decline in petal reflex was observed with increasing storage time, particularly after prolonged storage periods (≥40 days). This trend was more pronounced in cv. ‘Rhodos’, whereas cv. ‘Athena’ maintained a relatively higher opening capacity under similar conditions. These results suggest that cultivar-specific physiological characteristics play a major role in determining bud development responses during postharvest storage.

These findings are consistent with previous studies indicating that bud opening in cut roses is influenced by cultivar characteristics, storage conditions, and ethylene-related physiological processes [29,32,33]. In addition, controlled atmosphere conditions have been reported to delay petal reflex and extend vase life in cut flowers [34,35].

Recent studies have also highlighted that postharvest physiological status and stress-regulating mechanisms can significantly influence bud opening and overall flower quality in cut roses [12,26].

## 4. Conclusions

The results of this study clearly demonstrate that the postharvest performance of cut roses is strongly influenced by the interaction between cultivar characteristics, storage atmosphere, and applied postharvest treatments. Significant differences were observed between cvs. ‘Rhodos’ and ‘Athena’, confirming that cultivar-specific physiological responses play a decisive role in determining storage potential, quality preservation, and vase life.

Among the evaluated treatments, 1-methylcyclopropene (1-MCP) generally improved postharvest performance by maintaining chlorophyll stability, preserving color attributes, reducing *Botrytis cinerea* development, and regulating bud opening dynamics during vase life. In addition, controlled atmosphere conditions (CA10:3 and CA6:3) and LDPE-based modified atmosphere packaging contributed to quality preservation by moderating physiological activity and delaying senescence-related changes. However, the effectiveness of these treatments varied depending on storage duration and cultivar response.

The cultivar ‘Athena’ exhibited greater tolerance to prolonged storage, maintaining higher color stability, lower disease severity, and a more consistent bud opening capacity compared to ‘Rhodos’. In contrast, ‘Rhodos’ showed more limited petal reflex and greater susceptibility to physiological and pathological deterioration during extended storage. These findings highlight the importance of considering cultivar-specific behavior when optimizing postharvest handling strategies.

Furthermore, prolonged storage duration was identified as a critical factor affecting all quality parameters, particularly beyond 40 days, where reductions in physiological performance and increased disease susceptibility became more pronounced. This emphasizes the need to carefully balance storage duration with treatment strategies to maintain marketable quality.

Overall, the integration of 1-methylcyclopropene (1-MCP) with controlled atmosphere technologies and LDPE-based packaging systems may represent a promising approach for preserving postharvest quality in cut roses, although its effectiveness may vary depending on cultivar and storage conditions.

## Figures and Tables

**Table 1:** Effects of atmosphere × treatment combinations on SPAD values (V_0_, V_e_ and ΔSPAD) in cv. ‘Rhodos’.

Harvest Period	Atmosphere	Treatment	V_0_ (SPAD) ± SE	V_e_ (SPAD) ± SE	ΔSPAD	Tukey Group
May	CA10:3	1-MCP	51.4 ± 0.9	54.3 ± 1.0	+2.9	a
May	LDPE2	HS	52.3 ± 1.1	56.8 ± 1.2	+4.5	a
June	CA10:3	1-MCP	55.8 ± 1.3	60.8 ± 1.1	+5.0	a
August	LDPE1	1-MCP	58.2 ± 1.6	57.2 ± 1.5	−1.0	b
November	LDPE2	1-MCP	61.8 ± 1.4	56.7 ± 1.3	−5.1	c

V_0_: beginning of vase life; V_e_: end of vase life. ΔSPAD = V_e_ − V_0_. HS: hydrating solution (Chrysal RVB Clear Intensive, 1 mL L^−1^). Means followed by different letters indicate significant differences according to Tukey’s test at α = 0.01.

**Table 2:** Effects of atmosphere × treatment combinations on SPAD values (V_0_, V_e_ and ΔSPAD) in cv ‘Athena’.

Harvest Period	Atmosphere	Treatment	V_0_ (SPAD) ± SE	V_e_ (SPAD) ± SE	ΔSPAD	Tukey Group
May	CA6:3	1-MCP	27.9 ± 1.2	27.8 ± 1.3	−0.1	b
May	LDPE2	HS	28.3 ± 1.3	31.2 ± 1.5	+2.9	a
June	CA10:3	1-MCP	42.1 ± 1.4	40.5 ± 1.5	−1.6	b
August	LDPE1	1-MCP	47.8 ± 1.5	40.5 ± 1.6	−7.3	c
November	CA6:3	1-MCP	43.9 ± 1.3	43.8 ± 1.4	−0.1	b

V_0_: beginning of vase life; V_e_: end of vase life. ΔSPAD = V_e_ − V_0_. HS: hydrating solution (Chrysal RVB Clear Intensive, 1 mL L^−1^). Means followed by different letters indicate significant differences according to Tukey’s test at α = 0.01.

**Table 3:** Effects of atmosphere × treatment combinations on hue angle (h°) and chroma (C) values (V_0_, V_e_ and Δ) in sepal and petal tissues of cv. ‘Rhodos’.

Harvest Period	Treatment	h° V_0_	h° V_e_	Δh°	C* V_0_	C* V_e_	ΔC*
May	Control	−56.7	−39.2	+17.5	22.9	11.9	−11.0
May	HS	−55.9	−38.9	+17.0	21.3	11.5	−9.8
May	1-MCP	−57.4	−37.7	+19.7	33.6	11.8	−21.8
May	CA10:3 + 1-MCP	−41.5	−42.8	−1.3	6.3	5.4	−0.9
May	LDPE2 + 1-MCP	−43.1	−38.7	+4.4	6.5	6.5	0.0
June	Control	−41.0	−46.8	−5.8	5.0	6.2	+1.2
June	1-MCP	−42.3	−47.0	−4.7	5.0	5.9	+0.9
June	LDPE1 + 1-MCP	−41.6	−61.9	−20.3	4.3	5.6	+1.3
June	CA6:3 + 1-MCP	−41.5	−70.0	−28.5	3.8	8.2	+4.4
August	Control	−40.6	−51.8	−11.2	4.5	7.5	+3.0
August	1-MCP	−40.9	−57.4	−16.5	4.8	10.9	+6.1
August	LDPE2 + 1-MCP	−42.1	−43.9	−1.8	6.1	6.5	+0.4
November	Control	−31.4	−52.5	−21.1	4.7	6.3	+1.6
November	HS	−32.8	−44.5	−11.7	5.1	4.7	−0.4
November	1-MCP	−41.6	−32.3	+9.3	5.9	3.2	−2.7
November	LDPE2 + 1-MCP	−24.4	−26.5	−2.1	2.5	2.8	+0.3
November	CA6:3 + 1-MCP	−44.5	−53.2	−8.7	8.0	11.0	+3.0
November	CA10:3 + 1-MCP	−45.4	−66.0	−20.6	8.7	14.0	+5.3

V_0_: beginning of vase life; V_e_: end of vase life. Δ = V_e_ − V_0_. HS: hydrating solution (Chrysal RVB).

**Table 4:** Effects of atmosphere × treatment combinations on hue angle (h°) and chroma (C) values (V_0_, V_e_ and Δ) in sepal and petal tissues of cv. ‘Athena’.

Harvest Period	Treatment	h° V_0_	h° V_e_	Δh°	C* V_0_	C* V_e_	ΔC*
May	Control	−77.5	−87.5	−10.0	26.4	27.6	+1.2
May	HS	−76.6	−89.0	−12.4	26.2	28.9	+2.7
May	1-MCP	−74.5	−86.4	−11.9	24.1	27.2	+3.1
May	CA10:3 + 1-MCP	−59.5	−59.7	+0.2	36.7	35.0	−1.7
May	LDPE2 + 1-MCP	−47.9	−49.2	−1.3	11.1	21.1	+10.0
June	Control	−47.8	−46.4	+1.4	12.6	12.1	−0.5
June	1-MCP	−48.5	−47.9	+0.6	14.3	15.7	+1.4
June	LDPE1 + HS	−47.1	−50.6	−3.5	10.4	15.0	+4.6
June	CA6:3 + 1-MCP	−46.0	−43.6	+2.4	14.7	7.8	−6.9
August	Control	−45.1	−70.1	−25.0	7.3	10.9	+3.6
August	1-MCP	−44.8	−71.5	−26.7	7.7	10.1	+2.4
August	LDPE2 + 1-MCP	−42.8	−53.2	−10.4	6.0	11.3	+5.3
August	CA6:3 + 1-MCP	−42.8	−53.2	−10.4	6.0	11.3	+5.3
November	Control	−47.8	−85.0	−37.2	12.6	6.0	−6.6
November	HS	−42.9	−75.1	−32.2	10.4	6.0	−4.4
November	1-MCP	−48.5	−75.0	−26.5	14.3	6.1	−8.2
November	LDPE2 + 1-MCP	−35.3	−70.6	−35.3	4.4	5.4	+1.0
November	CA6:3 + 1-MCP	−72.0	−85.0	−13.0	14.5	18.4	+3.9

V_0_: beginning of vase life; V_e_: end of vase life. Δ = V_e_ − V_0_. HS: hydrating solution (Chrysal RVB).

**Table 5:** Effects of atmosphere × treatment combinations on *Botrytis cinerea* incidence (%) and severity (1–5 scale) in cvs. ‘Rhodos’ and ‘Athena’.

Harvest Period	Cultivar	Treatment	Incidence (%)	Severity (1–5)
May	Rhodos	Control	100 ± 0.0 a	4.5 ± 0.2 a
		1-MCP	95 ± 2.0 a	4.2 ± 0.3 a
		LDPE2 + 1-MCP	60 ± 3.1 b	2.8 ± 0.2 b
		CA10:3 + 1-MCP	55 ± 2.8 b	2.5 ± 0.2 b
May	Athena	Control	80 ± 3.5 a	3.9 ± 0.3 a
		HS	0 ± 0.0 c	0.0 ± 0.0 c
		LDPE1 + HS	0 ± 0.0 c	0.0 ± 0.0 c
		LDPE2	0 ± 0.0 c	0.0 ± 0.0 c
August	Athena	1-MCP	60 ± 2.8 b	2.0 ± 0.2 b
		LDPE2 + 1-MCP	55 ± 2.5 b	1.9 ± 0.2 b
November	Rhodos	1-MCP	90 ± 3.2 a	3.6 ± 0.3 a
		LDPE2 + 1-MCP	75 ± 2.9 b	3.0 ± 0.2 b
November	Athena	Control	100 ± 0.0 a	4.6 ± 0.3 a
		1-MCP	40 ± 2.1 c	1.2 ± 0.1 c
		LDPE1 + 1-MCP	45 ± 2.4 c	1.4 ± 0.2 c
		HS	50 ± 2.7 c	1.5 ± 0.2 c

Severity was evaluated using a 1–5 scale (1 = no visible infection, 5 = severe infection). Incidence (%) was calculated as the percentage of infected flowers within each treatment group. Values are presented as mean ± standard error (SE). Different letters within the same harvest period and cultivar indicate significant differences according to Tukey’s test (*p* < 0.01). Values of 0 indicate that no visible *Botrytis* infection was observed in any replicate within the corresponding treatment. HS: hydrating solution (Chrysal RVB). LDPE1 and LDPE2 represent low- and high-permeability polyethylene packaging materials, respectively. CA10:3 and CA6:3 denote controlled atmosphere conditions (10% CO_2_ + 3% O_2_ and 6% CO_2_ + 3% O_2_, respectively).

**Table 6:** Effects of atmosphere × treatment combinations on bud development (petal reflex score) in cv. ‘Rhodos’ (1–3 scale).

Treatment	May	June	August	November
NA + Control	2.27 ± 0.18 a	2.96 ± 0.04 a	2.88 ± 0.07 a	2.84 ± 0.06 a
NA + HS	2.22 ± 0.17 a	3.00 ± 0.00 a	-	2.92 ± 0.05 a
NA + 1-MCP	2.40 ± 0.19 a	2.93 ± 0.05 a	2.90 ± 0.05 a	2.92 ± 0.05 a
LDPE1 + Control	2.00 ± 0.18 a	2.82 ± 0.07 a	2.91 ± 0.05 a	2.85 ± 0.06 a
LDPE1 + HS	1.82 ± 0.14 a	3.00 ± 0.00 a	-	3.00 ± 0.00 a
LDPE1 + 1-MCP	2.00 ± 0.18 a	3.00 ± 0.00 a	2.91 ± 0.05 a	3.00 ± 0.00 a
LDPE2 + Control	2.14 ± 0.21 a	2.96 ± 0.04 a	2.85 ± 0.06 a	2.88 ± 0.05 a
LDPE2 + HS	1.97 ± 0.18 a	2.89 ± 0.06 a	-	2.91 ± 0.05 a
LDPE2 + 1-MCP	2.31 ± 0.23 a	2.89 ± 0.06 a	3.00 ± 0.00 a	2.93 ± 0.04 a
CA10:3 + Control	1.91 ± 0.16 a	-	-	-
CA10:3 + HS	1.89 ± 0.17 a	-	-	-
CA10:3 + 1-MCP	1.88 ± 0.16 a	-	-	-
CA6:3 + Control	2.06 ± 0.18 a	-	-	-
CA6:3 + HS	2.11 ± 0.20 a	-	-	-
CA6:3 + 1-MCP	2.17 ± 0.20 a	-	-	-

In cv. ‘Rhodos’, bud development was evaluated using a 1–3 scale (1 = closed bud, 3 = partially open flower). Values are presented as mean ± standard error (SE). Means followed by different letters within the same column indicate significant differences according to Tukey’s test at α = 0.01. -: not evaluated. HS: hydrating solution (Chrysal RVB). LDPE1 and LDPE2 represent low- and high-permeability packaging materials, respectively. CA10:3 and CA6:3 denote controlled atmosphere conditions.

**Table 7:** Effects of atmosphere × treatment combinations on bud development (petal reflex score) in cv. ‘Athena’ (1–5 scale).

Treatment	May	June	August	November
NA + Control	2.91 ± 0.24 b	3.50 ± 0.29 b	4.62 ± 0.14 a	3.67 ± 0.22 b
NA + HS	3.36 ± 0.25 ab	3.57 ± 0.21 b	-	3.64 ± 0.19 b
NA + 1-MCP	3.10 ± 0.25 ab	3.39 ± 0.24 b	4.19 ± 0.19 a	3.46 ± 0.18 c
LDPE1 + Control	2.42 ± 0.22 c	4.21 ± 0.12 a	3.68 ± 0.27 b	4.26 ± 0.13 a
LDPE1 + HS	2.79 ± 0.27 bc	3.61 ± 0.20 b	-	3.61 ± 0.20 b
LDPE1 + 1-MCP	2.85 ± 0.27 bc	4.21 ± 0.17 a	4.48 ± 0.18 a	4.04 ± 0.16 a
LDPE2 + Control	2.83 ± 0.27 bc	4.00 ± 0.17 a	3.64 ± 0.27 b	4.03 ± 0.16 a
LDPE2 + HS	2.83 ± 0.29 bc	3.50 ± 0.15 b	-	3.50 ± 0.15 b
LDPE2 + 1-MCP	2.92 ± 0.28 b	3.86 ± 0.16 ab	4.18 ± 0.17 a	4.09 ± 0.15 a
CA10:3 + Control	2.92 ± 0.27 b	-	-	-
CA10:3 + HS	2.90 ± 0.27 b	-	-	-
CA10:3 + 1-MCP	3.00 ± 0.28 b	-	-	-
CA6:3 + Control	2.95 ± 0.27 b	-	-	-
CA6:3 + HS	2.98 ± 0.28 b	-	-	-
CA6:3 + 1-MCP	2.83 ± 0.26 bc	-	-	-

In cv. ‘Athena’, bud development was evaluated using a 1–5 scale (1 = closed bud, 5 = fully open flower). Values are presented as mean ± standard error (SE). Means followed by different letters within the same column indicate significant differences according to Tukey’s test at α = 0.01. -: not evaluated. HS: hydrating solution (Chrysal RVB). LDPE1 and LDPE2 represent low- and high-permeability packaging materials, respectively. CA10:3 and CA6:3 denote controlled atmosphere conditions.

## Data Availability

The original contributions presented in this study are included in the article. Further inquiries can be directed to the corresponding author.
