# Correction: İnce et al. Controlled and Modified Atmospheres Combined with 1-MCP Improve Postharvest Quality and Suppress *Botrytis cinerea* in Cut Roses (*Rosa hybrida* L.). *Plants* 2026, *15*, 1452

**DOI:** 10.3390/plants15132057

**Published:** 2026-07-02

**Authors:** Ertürk İnce, Nuray Akbudak, Oktay İnce, Fisun Gürsel Çelikel

**Affiliations:** 1Atatürk Horticultural Central Research Institute, Yalova 77100, Türkiye; oktay.ince@tarimorman.gov.tr; 2Department of Horticulture, Faculty of Agriculture, Bursa Uludağ University, Bursa 16059, Türkiye; nakbudak@uludag.edu.tr; 3Department of Horticulture, Faculty of Agriculture, Ondokuz Mayıs University, Samsun 55139, Türkiye; fgcelikel@omu.edu.tr

## Addition of an Author

There are some corrections of the published manuscript [1].

Prof. Dr. Fisun Gürsel Çelikel was not included as an author in the original publication. Following post-publication evaluation, all authors agreed that her contributions to the conceptualization, investigation, methodology, project administration, and supervision of the study meet the journal’s authorship criteria. The authors apologize for any inconvenience caused.

The Author Contributions statement has been updated as follows: Conceptualization, E.İ. and F.G.Ç.; Methodology, E.İ., N.A., O.İ. and F.G.Ç.; Formal analysis, N.A. and O.İ.; Investigation, E.İ., N.A., O.İ. and F.G.Ç.; Resources, O.İ.; Data curation, E.İ.; Writing—original draft preparation, E.İ.; Writing—review and editing, E.İ., N.A. and O.İ.; Visualization, E.İ.; Project administration, N.A. and F.G.Ç.; Supervision, F.G.Ç. All authors have read and agreed to the published version of the manuscript.

## References

During the preparation of this correction, the reference list was carefully re-examined and bibliographic updates were made to References 10 and 23 to improve the accuracy and relevance of the cited literature.

Reference 10 has been updated from

Çelikel, F.G.; Reid, M.S. Postharvest performance of cut flowers under different temperature conditions. *Acta Hortic.*
**2002**, *543*, 145–152.

to

Çelikel, F.G. Preharvest and postharvest factors in sustainable quality management of ornamental plants. *Acta Hortic.* **2024**, *1397*, 15–22. https://doi.org/10.17660/ActaHortic.2024.1397.3.

Reference 23 has been updated from

Çelikel, F.G. Postharvest physiology of ornamental plants. *Acta Hortic.* **2013**, *1000*, 45–52.

to

Çelikel, F.G. Sustainable postharvest temperature and disease management of roses. IX International Symposium on Rose Research and Cultivation (ISRRC 2025). *Acta Hortic. in press*.

The authors state that the scientific conclusions are unaffected. This correction was approved by the Academic Editor. The original publication has also been updated.
